# The Feasibility of Animal-Based Indicators of Consciousness and Unconsciousness for Stunning in Sheep: A Systematic Review

**DOI:** 10.3390/ani13081395

**Published:** 2023-04-18

**Authors:** Marta Comin, Sara Barbieri, Michela Minero, Emanuela Dalla Costa

**Affiliations:** Department of Veterinary Medicine and Animal Sciences, Università degli Studi di Milano, 26900 Lodi, Italy; sara.barbieri@unimi.it (S.B.); michela.minero@unimi.it (M.M.); emanuela.dallacosta@unimi.it (E.D.C.)

**Keywords:** sheep, stunning, animal-based indicator, consciousness, unconsciousness, feasibility, slaughter, animal welfare

## Abstract

**Simple Summary:**

Animal welfare at slaughter is one of the most critical points raising moral questions of ethical sustainability. One way to reach the animal welfare requirements demanded by law would be to monitor the loss of consciousness in animals at the time of the killing to avoid unnecessary pain, stress, and fear. Animal-based indicators (ABMs), directly observed on the animal, are used to assess states of consciousness and unconsciousness, and thus the effectiveness of the stunning method. This is also true for sheep; however, there is still a lack of information on their application under the different conditions in which sheep are slaughtered. This study aimed to critically evaluate the aspects which can strongly affect the possibility of evaluating ABMs, beginning with a literature review of the scientific information available. The results outlined the need to deepen and cover information gaps on the feasibility of ABMs, since different physical aspects can affect the possibility of evaluating the indicators, and thus hamper the protection of sheep welfare at slaughter.

**Abstract:**

Background: According to EU legislation, animal-based indicators (ABMs) are used to evaluate the efficacy of stunning methods to ensure that animals do not regain consciousness. EFSA has provided a list of ABMs for electrical and mechanical stunning in sheep; however, there is still a lack of information on their feasibility. We aimed to identify and evaluate the feasibility constraints of ABMs commonly applied in slaughterhouses to assess proper stunning in sheep. Method: For this systematic review, we searched the Scopus and Web of Science databases from 2000 to 8 August 2022, including full peer-reviewed papers written in English on the welfare of sheep at the stunning and restraint phases. We excluded studies using a gas stunning method or without prior stunning, as well as manuscripts in which indicators were applied after sticking. Results: Of 1289 records identified, only 8 papers were eligible for the critical evaluation of physical aspects that affect the feasibility of ABMs. These aspects were defined as a given definition of the feasibility of ABMs, and information was summarized and critically evaluated. The results highlighted a lack of information on the feasibility of ABMs which should be considered in the various conditions of commercial slaughterhouses.

## 1. Introduction

The time of killing is a well-known stressful event in which animals can experience a high level of fear and pain [1,2]. To prevent unpleasant sensations, a temporary or permanent disruption of brain function can occur because of brain concussion, administration of anesthetics, anoxia, or an electroconvulsive shock [3]. This state is unconsciousness, and it is defined as the animal being unable to consciously perceive stimuli, including pain [4]. In Europe, the need to render the animals insensible and unconscious to prevent unnecessary suffering of animals is ensured by Council Regulation (EC) No 1099/2009 on the protection of animals at the time of killing. According to Article 4, animals must be rendered unconscious by the stunning method and remain so until death occurs through bleeding [5]. Stunning is the intentionally induced process which causes loss of consciousness and sensibility without pain, including any process resulting in instantaneous death [5]. The only exception is the practice of religious slaughter; nevertheless, after the publication of Press Release 163/20 of the EU Court of Justice, Member States can require a reversible stunning procedure which cannot result in the animal’s death [6]. Following the application of the stunning method, the insensibility state should be ensured and maintained before the shackling and hoisting of livestock to avoid unnecessary stress or pain [7]; therefore, those responsible for stunning must carry out regular checks directly on the animals between the end of stunning process and death [5]. Monitoring the absence of any sign of conscious sensibility is vital to guarantee animal welfare at the time of killing [8], and the most objective method is the evaluation of brain activity using an electroencephalogram (EEG). However, there are some disadvantages in the application of EEG during slaughtering that permit its use only under specific experimental conditions [9,10,11,12]. Verhoeven and colleagues (2015) clearly described the disadvantages in the use of EEG; for example, when the results are possibly influenced by animal- (eye or muscle movements) or technical-related (cable movements, impedance fluctuation, or 50/60 HZ interference) artefacts [13]. At slaughter, behavioral indicators of consciousness and unconsciousness are regularly used to monitor the insensibility of the animal after the stun [8,12,14,15,16,17,18,19]; this is true also for sheep [20]. In 2021, EFSA published an Opinion on the welfare of sheep at slaughter [16], identifying a list of nine valid animal-based measures (ABMs) (Table 1) commonly used to evaluate the effectiveness of stunning methods in sheep [14,15,16,21].

However, the precise relationships of ABMs with brain state, or with other indicators of correct or incorrect stunning, are insufficiently known and appear to depend on the type of stunning or slaughter technique used [8]. Furthermore, the interpretation of all individual ABMs may be questionable unless supported by other information [22,23], and it is highly recommended to use multiple indicators to assess and determine unconsciousness [12,18]. In the context of sheep slaughter, huge differences can be identified, starting from the stunning and restraint methods, which can vary considerably from one slaughterhouse to another. A specific list of stunning methods is available in Annex 1 of Council Regulation (EC) No. 1099/2009 [5]; for sheep, the main methods reported are the head-only electrical stun and the penetrative captive bolt stun. In the case of correct application of the stun, the main difference between the two methods is that the electrical is reversible, so the animal would regain sensibility after a certain time [9], while the mechanical leads to the death of the animal [24]. Another method considered by the EFSA AHAW Panel (2021) is head-to-body electrical stunning [16], an irreversible method which eliminates the possibility of recovery of consciousness. An effective head-to-body electrical stunning is characterized by the same indicators applied to the head-only electrical method [16].

The restraint methods available for sheep include the use of group stunning pens, where animals are grouped in a pen and an operator can restrict one animal at a time prior to stunning. Similar is the condition in which a single animal is taken from the unloading area and manually restrained, typical of small abattoirs. In other slaughterhouses, the restraining devices are specially designed, such as V-type restraining boxes, V-shape conveyors, and central track restrainers. In addition, the difference between industrial and small ‘familiar’ slaughterhouses can affect the stunning and bleeding operations. In small abattoirs, it is common to stun and bleed the animal in the same area, while in industrial abattoirs an automatic slaughter line brings the stunned animal in suspension from the shackle to the bleed area. The suspension on the line limits the movements of the animals [2], therefore also limiting the observability of ABMs.

In the literature, the studies on stunning of sheep focus more on the technical aspects of the procedures, such as the adequate electrical parameters or the correct positioning of the captive-bolt gun; the most recent results are clearly reported in the latest Scientific Opinion published by EFSA in 2021 [16]. Furthermore, most of the scientific information that investigated the consequences of stunning on responsiveness and brain activity of sheep is dated [22,25,26,27,28]. It is also necessary to update the information to follow the evolution of the technologies currently used in slaughterhouses. In 2013, EFSA published a Scientific Opinion reporting the performance (i.e., sensitivity, specificity, and feasibility) of the main indicators for electrical stunning in sheep in the phase between electrical stunning and the death of the animals [15]. The feasibility of the sheep indicators was based on expert opinions (Table 2), and has not been investigated from a practical point of view, excluding the identification of specific conditions which may limit the applicability of an indicator in a given context.

To date, no research has been performed to evaluate the applicability and feasibility of these behavioral indicators of consciousness and unconsciousness for sheep at the abattoir. Hence, we derived the definition of feasibility of behavioral indicators of stunning at slaughter from the one reported by EFSA, which includes different physical aspects of the animal-based measure (e.g., the position of the animal relative to the assessor, the assessor’s access to the animal and the line speed) [15]. Other important physical constraints are the distance between the animal and the assessor, the time needed for the evaluation, and the observational constraints in the animals (e.g., presence of blood in the eyes or convulsion/movements restricted by the restraint method). These physical constraints can differ between small and industrial slaughterhouses, as well as between electrical and mechanical stunning and different restraint methods (e.g., manually by an operator or in a group pen).

The aim of this review was to critically evaluate the feasibility and applicability of animal-based indicators used for determining the effectiveness of stunning and the unconsciousness of sheep at the time of killing.

## 2. Materials and Methods

A systematic literature search based on the Preferred Reporting Items for Systematic Reviews and Meta-Analyses (PRISMA) methodology [29] was conducted (the PRISMA checklist is reported in Supplementary Materials, Table S1). The literature search was conducted through Scopus and Web of Science on 8 August 2022.

The search terms were selected to identify the available literature on the welfare of sheep at slaughter, considering the restraint and stunning phases. Thus, the following search terms were included: (sheep OR lamb OR mutton OR ram OR “Ovis aries”) and (“welfare” AND slaughter OR stun* OR restrain*). Additionally, another search was conducted to focus on the use of animal-based indicators to assess consciousness and unconsciousness in sheep after stunning, using the following terms: (behavio* OR “animal-based” OR ABM OR indicator OR conscious* OR unconscious* OR feasib* OR effectiveness OR assess* OR EEG). To facilitate the selection process, the language and publishing period were set based on the inclusion criteria mentioned above.

Only full peer-reviewed papers written in English and published between 2000 and 8 August 2022 were included in the search. Since this critical evaluation focused on the stunning phase in sheep and lambs, all studies using gas stunning or without prior stunning (e.g., ritual slaughter) were excluded. Moreover, we excluded studies in which indicators were applied after the sticking of the animals, with the objective of identifying only results focused on assessing the state of insensibility of the animals immediately after stunning. A paper was considered eligible for inclusion in the results if the author collected information on the application of at least one of the ABMs used at stunning in sheep as a part of a qualitative, observational, or experimental study.

All papers obtained from the database searches were exported into a CSV file (Microsoft Excel), and a first cleaning of the dataset was conducted to maintain only peer-reviewed papers and to remove papers that did not report the authors’ names or abstract. Before screening, all duplicates were removed. The screening process was carried out by the first author (M.C.). Firstly, any record with a title that clearly did not fit the eligibility criteria was excluded. The remaining papers were screened by the first author (M.C.) based on the abstract, and subsequently on the full text. The information regarding the study environment, animal category, stunning method, restraint method, and physical aspects of feasibility of the ABMs used in the studies to assess the efficacy of the stunning method were extracted; the derived information was handled and tabulated in tables (Table 3 and Table 4).

The definition of feasibility of animal-based indicators of stunning at slaughter was derived from the one reported by EFSA, which includes different physical aspects of the animal-based measure assessment (e.g., the position of the animal relative to the assessor, the assessor’s access to the animal, and line speed) [15]. Other aspects were then considered, such as the distance between the assessor and the animal, the time needed to evaluate the indicator together with the possibility of assessing the indicator immediately after stunning (in accordance with the formal recommendations on the duration of the stun-to-stick interval versus the time required to assess the indicator), the use of specific tools, and observational constraints (i.e., presence of blood in the eyes of the assessed animal, blind spots, and movements during the shackling and hoisting procedures).

For each indicator, all available information was summarized into tables to identify: the study environment (e.g., experimental or under field conditions), the animal category (lambs or sheep), the stunning method applied (e.g., mechanical or electrical), the restraint method (i.e., individual or group stunning pens), the time needed to assess the measure, the position of the assessor relative to the animal, the distance between the assessor and the animal, access to the animal, the use of specific equipment for the evaluation, and observational constraints (Table 3 and Table 4).

## 3. Results and Discussion

**Figure 1:**
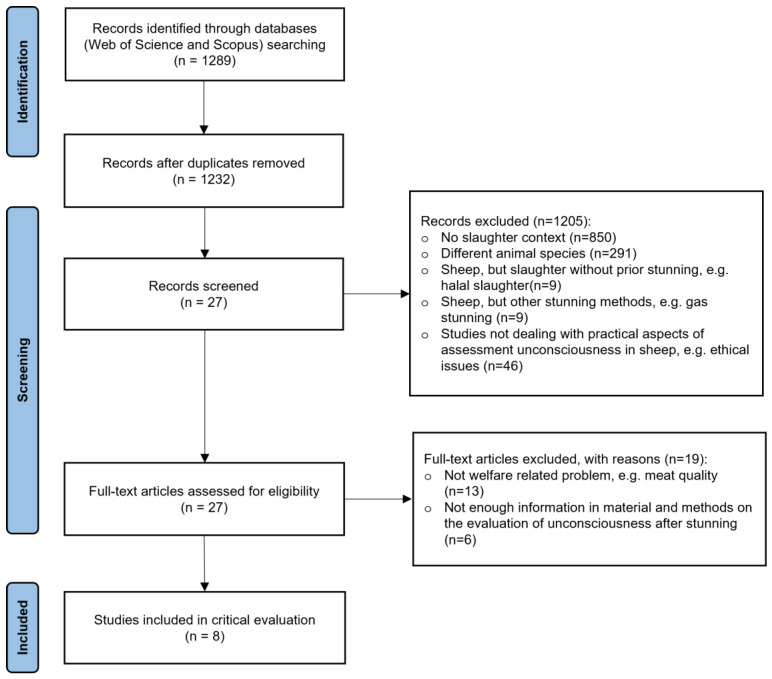
Modified Preferred Reporting Items for systematic Reviews and Meta-Analyses (PRISMA) flow diagram [29] with the review search strategy and study selection.

The literature search resulted in 1289 records. After duplicate removal and the application of exclusion criteria throughout the review process, a total of 1281 papers were omitted. A modified PRISMA flow diagram provides information on the number of excluded papers and the reason for exclusion (Figure 1). The full dataset derived from the literature search is presented in Table S2 (Supplementary Materials).

We identified a total of eight papers relevant to the critical evaluation of the physical aspects of the feasibility of animal-based indicators of stunning in sheep. They included information on the application of eight animal-based indicators. Most studies were conducted under experimental conditions, including experimental abattoirs (*n* = 5); two studies were conducted on-farm (*n* = 2), whilst only one study was conducted at an abattoir (*n* = 1). Only one study was focused on adult sheep (*n* = 1), and the age of lambs included in the studies (*n* = 7) ranged from birth to 12 months. One study was conducted on anaesthetized lambs, one study with a non-penetrative captive bolt on neonatal lambs, and one study with a penetrative captive bolt, while in the other 5 studies the stun method used was the electrical head-only. Only two studies reported a stun-to-stick interval within 20 s. Most restraint methods were designed for the experimental nature of the study, while in only three studies the method was consistent with those commonly used in abattoirs. Breathing and corneal reflex were the most reported animal-based indicators (*n* = 8 and *n* = 7 respectively), followed by tonic–clonic convulsion (*n* = 6), palpebral reflex (*n* = 3), muscle tone and eye movements (*n* = 2), and posture (*n* = 1); no study considered the presence or absence of vocalizations in sheep. At least two animal-based indicators were measured to determine insensibility after the stun. Vocalizations were not reported in any of the studies considered, hence they have not been discussed. No studies reported information on the position of the animal relative to the assessor, the distance between the assessor and the animal, or the presence of possible constraints during the assessment. Reviews, guidelines, book chapters, and European Opinions on the topic were used to discuss the results. The results are presented and discussed separately for each indicator (Table 3 and Table 4).

### 3.1. Breathing

Breathing can be assessed immediately after the stun as the absence (apnea) [20,30,33,34] or presence and return of rhythmic breathing [9,31,32,33,35]. After the stun, the immediate onset of apnea is a clear sign of unconsciousness or death, while the presence of breathing may not indicate a state of consciousness [20].

The return of spontaneous breathing after an effective head-only electrical stun is around 29 s (29.5 ± 1.55 s) if the animals are not exsanguinated [9], while, with a penetrative captive bolt, the animal dies within 10 min without exsanguination, following respiratory arrest which begins immediately after head impact [24]. In lambs, breathing was reported to be useful in assessing correct stunning by both head-only electrical [9,31,32,34,35] and non-penetrative captive bolt [30]. Verhoeven and colleagues (2015) used Propofol anesthesia as a model to validate indicators in the evaluation of unconsciousness. Only one study was performed on adult sheep using the penetrative captive bolt [33]. Six out of eight studies indicated that the presence of rhythmic breathing was assessed by evaluating the movements of the flank due to inhalation and exhalation [9,31,32,34,35]. However, they do not report other important information, such as distance from the animal, the position of the assessor with respect to the animal, the time needed to evaluate the indicator, and possible constraints. Furthermore, it is difficult to understand whether the type of restraint could influence the evaluation of this indicator, as previously reported for other species, such as calves [36]. When the animal is stunned in a group pen [32] or restrained manually by the operator [33], different positions of the animal and the distance between the animal and the assessor could result in different evaluations of the breathing state. Moreover, the use of a restrainer specially designed for experimental studies (i.e., net restrainer or custom-made hammock) [9,20,34,35] does not provide the necessary information to assess the feasibility of this ABM. In the online survey by the EFSA AHAW Panel (2013) [15] (Table 2), experts highlighted that it may be not possible to recognize breathing in animals in the stunning box during tonic–clonic seizures, and occasional or irregular gagging may occur in an unconscious animal just before the onset of brain death [12]. In addition, after the stun, the animals are handled and prepared for bleeding. Hence, we need to consider the difficulties of detecting movements of the flank during the subsequent processes. Velarde and colleagues (2000, 2002) reported the possibility of evaluating the movements of the flank using one lateral video camera. The video recordings were analyzed in correlation with EEG data collected on the same animal to confirm the status of unconsciousness. However, the use of video recordings under experimental conditions does not fit with the evaluation by direct observation of ABMs at slaughter. To evaluate the absence of rhythmic breathing, Velarde and colleagues (2000, 2002) also used breathing sounds, heard using a frontal video camera, while in another work [34] they did not specify the use of technological equipment. It is difficult to understand if the environmental conditions (i.e., noise originating from the processing line and operators) could disturb the evaluation of breathing sounds. In addition, they did not report how to assess the breathing sounds from the animal, whether there is need for direct access to the animal, or the maximum distance that allows recording of the sound. Llonch et al. (2015), in a study conducted in an experimental abattoir with animals stunned in a V-type restraining box, used a mirror to recognize the condensation under the snout. A mirror could be a very economic and easy-to-use instrument to help the assessor during the evaluation. However, to be suitable for an on-abattoir evaluation, information is needed regarding the shape and dimension of the mirror, or how to use it under the snout of the sheep. A mirror is a fragile object which may break during slaughtering operations, and it may not be kept in the correct position in relation to the animal. Moreover, environmental light can affect its use and mask the presence of condensation due to breath. Finally, Verhoeven and colleagues (2015) used a respiratory waveform recorded by an inductive respiratory band; however, placing this type of device around each animal’s abdomen is not suitable for the flow of the slaughter line; moreover, inappropriate handling of the animal could increase the level of fear [16]. To correctly assess the absence of breathing, Berg et al. (2012) suggested identifying at least two breaths by observing the movement of the flanks [19]. Considering the breathing frequency of lambs (15–25 breaths/min) [37], it is possible to deduce that approximately four to eight seconds are needed to assess the presence of the indicator. This time is in line with the recommended stun-to-stick interval. Von Holleben [38], in a document written for the training of German slaughter operators, suggests observing the presence of movements of the nostrils and mouth to evaluate the breathing. This method could be suitable and easy to apply when the animal is hanging from the slaughter line, with no convulsion, and the assessor could focus on the head. However, further research is needed to investigate this evaluation method under field conditions. In brief, there is still a lack of information on the feasibility of the absence of breathing as an indicator of a proper stun. Further research is needed to identify all physical aspects (i.e., the distance between the assessor and the animals, presence of convulsions, etc.) which can affect the evaluation of apnea along the flow of the slaughter line, from different types of restraint to evaluation methods.

### 3.2. Posture

Posture can be assessed as the immediate collapse or loss of posture in effectively stunned animals which are not restrained or prevented from doing so, while an ineffective stun can lead to the failure to collapse or attempts to regain posture after collapse [33]. A standing animal at slaughter is clearly conscious, while the loss of standing posture could indicate the loss of consciousness [8]. A correct and effective electrical stunning induces immediate collapse of the animal, as a result of the onset of tonic seizure [15]. Even in the case of mechanical stunning, after the shot animals should collapse immediately due to the damage to the reticular formation, which plays an important role in maintaining posture [39,40], and there are significant associations between incomplete concussion and failure to collapse [33]. Only one study reported the use of posture as an indicator of good stun quality on adult sheep stunned with a penetrative captive bolt [33]. The work of Gibson and colleagues (2012) was conducted on-farm on 489 adult sheep, which were penned individually with no restraint and shot, so the animals were free to collapse or show signs of recovery of posture. Immediately after shooting, the presence/absence of collapse was monitored for five minutes, or until the onset of cardiac arrest [33]. No additional information was reported, such as the distance between the animal and the assessor or the position of the assessor relative to the animal, even though the collapse is a clear and visible indicator directly observed on an animal, requiring no special equipment. The restraining of animals could prevent collapse, thus affecting the possibility of observing this indicator. Collapse cannot be assessed in animals stunned in V-shaped restrainers or conveyors [8,36], or if the animal is maintained in a resting position on the ground. Finally, it is important to consider the possibility of observing the indicator in relation to the conditions in which sheep are slaughtered, namely when animals are manually restrained in a standing position or using group pen stunning. Moreover, this indicator should be interpreted with caution; collapse can be caused by an inability to stand, for example, when the stun gun is placed in the neck and severs the upper spinal cord, only paralyzing the animal without damaging the brain [8].

### 3.3. Tonic–Clonic Seizure

Effective head-only electrical stunning can be assessed by tonic immobility during exposure to the stunning current, which lasts for several seconds and is followed by clonic seizures leading to loss of muscle tone [9,32,34,35,36]. In addition, after captive-bolt stunning, the animals express a tonic spasm prior to relaxation, with the following possibility of excessive convulsions [30].

Electrical head-only stunning induces tonic and clonic seizures, which are the outward symptoms of generalized epilepsy [15]. If the animal does not experience generalized epilepsy, the absence of tonic–clonic seizures is a clear indicator of failed stunning and possible consciousness [2]. However, after captive bolt stunning, the absence of a tonic spasm may be a sign of a low depth of concussion [2]. Velarde and colleagues (2002) defined the onset and duration of the tonic phase followed by two clonic phases. The onset of the tonic phase is immediately after collapse; it lasts an average time of 10.2 ± 0.29 s and is followed by two clonic phases, which generally manifest as kicking of legs, paddling, or galloping movements [9,15,26,34,35]. The first clonic phase (10.2 ± 0.29 s to 36.4 ± 1.23 s) is characterized by involuntary running movements of the hindlegs and extension, with very slight paddling movements, of the forelegs. The following second clonic phase (35.0 ± 1.11 s to 70.4 ± 1.79 s) involved more intense and less coordinated kicking of both the forelegs and hindlegs [9].

Scientific studies reported the presence of tonic–clonic seizures after electrical [9,31,32,34,35] and mechanical stunning in lambs [30]. Only one study was conducted under field conditions [32]. Similar to breathing, feasibility information on the distance of the assessor from the animal, the position of the assessor relative to the animal, the time needed to evaluate the indicator, and possible constraints were not reported. Furthermore, it is difficult to understand how the restraint method could affect the evaluation. In the online survey by the EFSA AHAW Panel (2013), experts rated the tonic seizure as easy or normal to assess at stunning, considering its absence as a sign of consciousness (Table 2). The general recommendation is to proceed with the bleeding during the tonic phase [2,14,15,16,17], before the onset of convulsions. Only one study focused on the presence or absence of any substantial kicking during the tonic phase [32]. The work was performed at an abattoir and the lambs were bled within 20 s, but a lack of information on the definition of “substantial kicking” makes the evaluation of this indicator difficult to understand and repeat. It is important to define a standard rate and train assessors to avoid subjective interpretation of the indicator. Five out of eight studies focused on the assessment of the intensity of the clonic phase [9,30,31,34,35], using different scores. However, the animals should be bled within a maximum of 15 s (8 s for head-only electrical stun [14]) to respect the general recommendations of the minimum stun-to-stick interval and avoid the recovery of sensibility. Hence, scoring of the clonic phase should not be needed. Velarde and colleagues (2000, 2002, 2003) subjectively scored the convulsion of each animal (1 = no to moderate movements; 2 = severe movements), demonstrating the same standardization problem mentioned above. In the first two works, they also used a lateral video camera and correlated brain activity using an EEG [9,35]. The use of video recordings and EEG activity to evaluate the indicator a posteriori may be a good method under experimental conditions; nevertheless, it cannot be used at slaughter. Similarly, Llonch and colleagues (2015) defined the intensity of clonic convulsions more specifically, with a description of the body parts involved in each category; hence, the subjective score could be suitable for assessor training. However, in this study, they focused on the presence of general tonic and clonic muscular activity, without specifying the observation time. Considering the average stun-to-stick intervals reported in the study (within 8 s), we can deduce that the evaluation of the clonic phase was performed in the post-cut period. Only one study was conducted on mechanical stunning by Grist and colleagues (2018), which defined three categories of clonic activity: mild, moderate, and severe uncontrolled physical movements. However, it is very difficult to standardize the descriptions of “coordinated” or “uncontrolled” movements [41,42]. In addition, to the need to fill the gaps in the feasibility aspects of the evaluation of this indicator, we need to deepen our knowledge of the patterns of tonic rigidity, with eyes wide open and rhythmic breathing absent [2], and of clonic convulsions, such as the physical differences between the two phases [9]. Velarde and colleagues (2002) reported the tonic phase in electrically stunned lambs as a rigid position with flexion of both the forelegs and hindlegs during current flow, while, at the end of the stun, an extension of the forelegs and head occurred. This allows recognition of which movements are inducted by epileptiform activity caused by stunning.

### 3.4. Corneal Reflex

The corneal reflex can be assessed by lightly touching or tapping the cornea of stunned animals [9,30,31,32,33,34,35]. Ineffectively stunned animals, and those recovering consciousness, will blink in response to the stimulus. However, unconscious animals may also intermittently show a positive corneal reflex. The absence of the corneal reflex is associated with loss of brain stem function [11], and thus loss of consciousness. However, there could be exceptions due to small focal lesions following hemorrhage [8] which can abolish the corneal reflex. For this reason, it is important to associate the absence of the corneal reflex with other indicators of unconsciousness [8]. The time to return of the corneal reflex in sheep after an effective head-only electrical stun is around 38 s (38.5 ± 1.75 s), coinciding with the end of the first clonic phase [9]; there is no similar information for the mechanical stun.

Seven out of eight studies assessed the corneal reflex, both on lambs and sheep, with mechanical and electrical stunning [9,30,31,32,33,34,35]. The method used to perform the corneal test was the same in all the studies, through physical stimulation of the cornea, which required the assessor’s access to the animal. Most of the studies were conducted under experimental conditions, so it was possible to touch the animal during the experiment, while the same information is missing under field conditions. In the online survey by the EFSA AHWA Panel (2013), several experts considered the corneal reflex as difficult to assess at stunning, and one of the reasons named was the inaccessibility of animals (Table 2). Nonetheless, it is essential to note the other missing information on the physical aspects of its assessment, with particular regard to the time which it takes to evaluate the corneal reflex after stunning. No studies reported the time during which the test was performed, nor if the test was performed using a finger or a small object such as a pen. During tonic seizure, the eyes should be wide open, with no response when touched [14]. Grandin (2013) suggested waiting to check the corneal reflex after electrical stunning, because, during the tonic phase, the reflex is blocked. Furthermore, in sheep, it is recommended to use a pen for the evaluation, due to their small eyes [19], and the corneal reflex test is difficult to interpret when the animal has blood in its eyes [8]. After electrical stunning, the vigorous movements of the clonic phase may interfere with the eyelid response, or make it difficult to carry out the test or interpret the results correctly [19].

In the context of slaughter, it is necessary to take into account the specific slaughter procedures and plants, in order to define when, where, and how to evaluate the corneal reflex along the slaughter line.

### 3.5. Palpebral Reflex

The palpebral reflex is elicited by touching or tapping a finger on medial canthus of the eye or eyelashes [20,30,33]. The corresponding neural circuit is largely similar to that of the corneal reflex [12]. Correctly stunned animals will not show a palpebral reflex, which results in the blinking of the eyelids. Ineffectively stunned animals, and those recovering consciousness, will blink in response to the stimulus. The palpebral reflex disappears earlier than the corneal reflex in anaesthetized animals [20], but there is no information on its feasibility under slaughter conditions in sheep after stunning.

Three out of eight studies reported the evaluation of palpebral reflex on sheep with propofol anesthesia [20], or sheep [33] and lambs [30] with mechanical stunning. Only the work of Verhoeven and colleagues (2015) described how to assess this indicator, while in the other studies no definition was reported, probably due to the similar evaluation of the corneal reflex. In conclusion, this similarity allows us to identify the same critical points in the evaluation of the feasibility of this indicator in slaughterhouses, such as the time and point where it is tested on stunned animals along the slaughter line.

### 3.6. Spontaneous Blinking

Spontaneous blinking is recognized when an eye makes a fully open-and-close cycle, as would live animals in the lairage, without touching the eye [18], and is a clear sign of consciousness, which involves circuits in the brainstem and the cortex [43]. Therefore, the presence of natural blinking may be used to recognize ineffective stunning or recovery of consciousness, especially if occurring with eye movements focused on external stimuli [2].

The result of the questionnaire on expert opinions [15] highlighted that spontaneous blinking could be difficult to assess due to inaccessibility to the animal (Table 2). Only one study assessed the presence of spontaneous blinking, by visual inspection, after head-only electrical stun on lambs restrained in a V-type restrainer box in an experimental abattoir [31]. It is a point of interest that the average stun-to-stick interval was within 10 s; hence, the sticking could be performed during the tonic phase of muscular rigidity. If the animal did not show any signs of recovery, such as the return of rhythmic breathing or spontaneous blinking, until the time of bleeding, it is possible to deduce that the stun-to-stick interval is a feasible time to assess this indicator when unprovoked, by visual-only inspection. The state of unconsciousness was also confirmed by the analysis of brain activity (EEG). However, visual inspection requires that the assessor constantly observes the animal until bleeding and death, so the assessor should be free from other activities and focused on the animal’s eyes, without performing any other test and without the presence of observational constraints (i.e., presence of convulsion or blood in the eyes). Grandin (2013) suggested the need to access the animal to evaluate spontaneous blinking in response to a hand waved in front of the eye, and to check this indicator within five seconds after stunning; these recommendations, together with further research aimed at filling in the missing information on the physical aspects of feasibility, will be useful for the correct evaluation of this indicator at stunning.

### 3.7. Muscle Tone

Muscle tone can be evaluated as a sign of unconsciousness by completely relaxed legs, floppy ears and tail, and relaxed jaws with protruding tongue [16], as well as no attempts to recover a normal posture [8]. On the other hand, ineffectively stunned animals and those recovering consciousness will show a righting reflex [30] and attempts to raise the head [32]. In the online survey by the EFSA AHWA Panel (2013), all experts scored the feasibility of the loss of muscle tone as easy to use at stunning (Table 2), also reporting that the animals which regain muscle tone may show stiff ears and jaws and the righting reflex (e.g., severe kicking, head lifting, or body arching). This was confirmed by the slaughter operator’s training document, in which Dr von Holleben identified movements of the ears as a warning signal which required the re-stun of the animal [8,38]. Gibson et al. (2012) evaluated the righting reflex and jaw muscle tension in sheep as parameters of good quality of mechanical stun, but no useful information about their evaluation was reported to assess their feasibility. The sheep were shot on-farm with no restraint; hence, we can deduce that the animals were free to collapse and regain posture in case of recovery. However, the animals in the study were not included in the routine slaughter process. For this reason, the observational constraints, and possible confounding movements due to the slaughter operations, are unknown. Berg et al. (2012) also evaluated the presence or absence of the head-righting reflex in a group pen of electrically stunned lambs which were stuck within 20 s of the stun. No information about the minimum distance or position of the assessor relative to the animal was reported. Probably, considering the type of restraint, the observation of the indicator was clear until the operation of hoisting and shackling the lambs. When hung on the rail, the head-righting reflex can be observed as arching of the back and sustained backwards lifting of the head. This should not be confused with a momentary flop of the head, which occurs when the back legs exhibit reflexive kicking [18], typical of the clonic phase [9]. Even then, due to the observational constraints of an animal restrained in a stunning box or moving conveyor, the evaluation of this indicator could be hindered [36]. It is often assessed when animals are removed from the stunning box or are hung on the bleeding rail [12]. This reflex is also difficult to assess when animals exhibit convulsions or involuntary body movements [27,28], and is difficult to distinguish from oriented movements of recovery [8]. Information on the feasibility of muscle tone as an indicator of proper stun needs to be refined with further research, focusing on the less confounding movements visible when the animals are hanging from the bleed rail or maintained in a stunning box, such as coordinated attempts of the animals to return to a normal posture and the movements of the ears.

### 3.8. Eye Movements

Eye movements can be evaluated by the presence of nystagmus (spontaneous rapid side-to-side movements of the eyeballs) or rotation of the eyeball [33], which can indicate ineffective stunning, while effectively stunned animals will exhibit fixed eyes (eyes wide open and glassy) [32] and pupil dilatation [33]. The expert opinions questionnaire reported the difficulty or impossibility of observing eye movements because of the orientation of the animal during the slaughter procedures [15] (Table 2). Berg and colleagues (2012) did not provide details on the evaluation of the eye movement indicator, but they considered the presence of both eyes coordinated and fixed on an object a sign of poor-quality stun. The head-only electrical stun was performed in a group pen with a stun-to-stick interval within 20 s. Hence we could deduce that, during this time, the assessor could observe, from a good distance, the eyes of the stunned animals until the sticking. This missing information, together with the unknown position of the assessor, needs to be covered. It is essential to note whether it was possible to focus on the eyes of the animal for the entire duration of the stun-to-stick interval. Gibson and colleagues (2012) evaluated, for five min after PCB, stun the presence or absence of the following indicators: eyeball rotation, nystagmus (vibrating eyelids), and pupil dilatation. It is known that nystagmus depends on the slaughter method used. Rolled back or vibrating eyes could be a sign of return to sensibility after a mechanical stunning, while it is possible to see nystagmus during the epileptic fit following effective electrical stunning [2]. Moreover, nystagmus could be confused with natural blinking if the assessor is not trained to recognize how conscious animals blink [19]. There was no restraint and no other information on possible observational constraints (e.g., blood from the point of impact on the head). The animals were not hung or stuck after the stun; hence, it is difficult to compare with typical slaughter conditions. Finally, it might be helpful to consider the indicator “eye movements” as single units, such as “eyeball rotation”, “nystagmus”, and “fixed eyes”, which could result in more specific further research aimed at deepening our knowledge of different physical aspects of feasibility with varying methods of slaughter in different slaughter contexts.

### 3.9. General Discussion

The critical evaluation of the feasibility of animal-based indicators at the abattoir highlighted specific areas where knowledge is lacking; in particular, we found that recent studies focused, rather than on ABMs, on the technical aspects of stunning methods, such as voltage and duration of application [31,32], and factors [35] that affect the quality of head-only electrical stunning or the correct position and pressure of the shoot for the mechanical method [33]. The method of stunning with gases was also investigated, assessing aversion phenomena with different gas concentrations [44]. In particular, this stunning method could allow the evaluation of different behavioral indicators which can be observed on free animals with no physical restraint, such as posture and seizures; hence, in a more controlled environment. When working in a slaughter scenario, it is paramount to consider the plurality of contexts in which the animal-based indicators must be applied to verify the correct stunning of the sheep, considering how the stunning method and restraint can affect their presence and observability.

Each year a large number of sheep are slaughtered, both in small abattoirs, with manual restraints, and industrial abattoirs, with V-shaped mobile conveyors in the processing line; thus, the observational and physical constraints may be very different. This plurality of contexts has certainly made it more difficult to assess the feasibility of these indicators than in the past. Furthermore, a study design based on anaesthetized animals does not allow the assessment of the feasibility of animal-based indicators in the commercial context [20], where the state of consciousness is evaluated by means of the presence of some physiological reflexes [2].

## 4. Conclusions

This review highlighted specific areas where information on the feasibility of animal-based indicators, routinely applied at slaughter, is lacking; these results confirm that the use of a single indicator may be doubtful and insufficient in assessing the efficacy of stunning [12]. The combined use of multiple indicators of consciousness and unconsciousness is strongly recommended to improve the evaluation of stunning at slaughter, and these may vary by applicability depending on different stunning methods [12]. Additionally, not all ABMs are applicable or relevant under all slaughter locations and conditions; hence, clarification of whether and how to apply current ABMs to confirm effective stunning could minimize the risk of consciousness at the time of bleeding. Further studies are needed to identify the minimal conditions (e.g., the position of the assessor relative to the animal, the distance between the assessor and the stunned animals, and the time required for each indication) required to properly assess the indicators of unconsciousness suggested by the EFSA AHAW Panel (2021) [16], singly or in combination.

## Figures and Tables

**Table 1:** Animal-based measures (ABMs) used to assess proper electrical and mechanical stunning in sheep *.

ABMs	Description	Stunning Method
breathing	Effective stunning will result in the immediate onset of apnea (absence of breathing). Ineffectively stunned animals and those recovering consciousness will start to breathe in a pattern commonly referred to as rhythmic breathing, which involves a respiratory cycle of inspiration and expiration. Rhythmic breathing can be recognized from regular movement of the flank and/or mouth and nostrils.	captive bolt/electrical
posture	Effective stunning will result in immediate collapse or loss of posture in animals that are not restrained or prevented from doing so. Ineffectively stunned animals, on the other hand, will fail to collapse or will attempt to regain posture after collapse.	captive bolt/electrical
corneal reflex	The corneal reflex is elicited by touching or tapping the cornea. Ineffectively stunned animals and those recovering consciousness will blink in response to the stimulus. Unconscious animals may also intermittently show a positive corneal reflex.	captive bolt/electrical
palpebral reflex	The palpebral reflex is elicited by touching or tapping a finger on the inner/outer eye canthus or eyelashes. Correctly stunned animals will not show a palpebral reflex. Ineffectively stunned animals and those recovering consciousness will blink in response to the stimulus.	captive bolt/electrical
eye movements	Eye movements, including nystagmus (spontaneous rapid side-to-side movements of the eyeballs) or rotation of the eyeball indicate ineffective stunning, as effectively stunned animals will exhibit fixed eyes.	captive bolt
vocalizations	Conscious animals may vocalize (bleating in goats and vocalization in lambs (Goldberg, 2018), and therefore purposeful vocalization can be used to recognize ineffective stunning or recovery of consciousness after stunning. However, not all conscious animals may vocalize.	captive bolt/electrical
spontaneous blinking	Conscious animals may show spontaneous blinking, in which the animal opens/closes eyelid on its own (fast or slow) without stimulation; therefore, this sign can be used to recognize ineffective stunning or recovery of consciousness after electrical stunning. However, not all the conscious animals may show spontaneous blinking.	captive bolt/electrical
tonic–clonic seizure	Effective head-only electrical stunning leads to the onset of tonic–clonic seizures soon after immediate collapse of the animal. The tonic seizure, which may be recognized from the tetanus, lasts for several seconds and is followed by clonic seizures lasting for seconds and leading to the loss of muscle tone.	electrical
muscular tone	Stunned animals will show general loss of muscle tone coinciding with the recovery of breathing and the corneal reflex if not previously stuck. Loss of muscle tone can be recognized from completely relaxed legs, floppy ears and tail, and relaxed jaws with protruding tongue. Ineffectively stunned animals and those recovering consciousness will show a righting reflex and attempts to raise the head.	captive bolt

* Table adapted from EFSA, Scientific Opinion (2021) [16].

**Table 2:** Feasibility of ABMs in head-only electrical stunning of sheep. Table based on data from the questionnaire of expert opinions of the EFSA AHAW Panel (2013) [15].

ABMs	Outcome	Feasibility	Feasibility Score ^1^
Breathing		TESTED questionnaire (*n* = 20)	
	Apnea.	easy: 40%	0.25
		normal: 45%	
		TESTED questionnaire (*n* = 16)	
Posture	Immediate collapse.	easy: 88%	0.81
		normal: 6%	
		TESTED questionnaire (*n* = 10)	
Corneal Reflex	No corneal reflex.	easy: 40%	0.2
		TESTED questionnaire (*n* = 4)	
Palpebral reflex	No palpebral reflex.	easy: 25%	0
		normal: 50%	
		TESTED questionnaire (*n* = 9)	
Eye movements	Fixed eyes (eyes wide open and glassy).	easy: 34%	0.11
		TESTED questionnaire (*n* = 12)	
Vocalizations	Presence of vocalization.	easy: 83%	0.83
		normal: 17%	
Spontaneous blinking		TESTED questionnaire (*n* = 8)	
	Presence of natural blinking.	easy: 50%	0.57
Tonic–clonic seizures		TESTED questionnaire (*n* = 16)	
	Absence of tonic seizure.	easy: 69%	0.69
		normal: 31%	
Muscle tone		TESTED questionnaire (*n* = 7)	
	Loss of muscle tone (floppy ears and relaxed jaws).	easy: 100%	1

^1^ Feasibility score = (number of = ‘easy’ respondents − number of ‘difficult’ respondents)/(number of all respondents).

**Table 3:** Critical evaluation of the feasibility of ABMs derived from the literature review—Study Environment. For each study, we report the study characteristics.

ABMs	References	Study Environment	Animal Category	Stunning Method	Restraining Method
Breathing	[30]	F	L	N-PCB	NO
	[20]	E	L	AN	NO
	[31]	ES	L	HO	VB
	[32]	S	L	HO	G
	[33]	F	S	PCB	NO
	[34]	ES	L	HO	NO
	[9]	E	L	HO	NO
	[35]	E	L	HO	NO
Posture	[33]	F	S	PCB	NO
Tonic–clonic seizure	[30]	F	L	N-PCB	NO
	[31]	ES	L	HO	VB
	[32]	S	L	HO	G
	[34]	ES	L	HO	NO
	[9]	E	L	HO	NO
	[35]	E	L	HO	NO
Corneal reflex	[30]	F	L	N-PCB	NO
	[31]	ES	L	HO	VB
	[32]	S	L	HO	G
	[33]	F	S	PCB	NO
	[34]	ES	L	HO	NO
	[9]	E	L	HO	NO
	[35]	E	L	HO	NO
Palpebral reflex	[30]	F	L	N-PCB	NO
	[20]	E	L	AN	NO
	[33]	F	S	PCB	NO
Spontaneous blinking	[31]	ES	L	HO	VB
Muscle tone	[32]	S	L	HO	G
	[33]	F	S	PCB	NO
Eye movements	[32]	S	L	HO	G
	[33]	E	S	PCB	NO

Study features: Study environment: E, experimental; ES, experimental slaughterhouse; F, farm; S, slaughterhouse. Animal category: L, lamb; S, sheep. Stunning method: AN, anesthesia; HO, head-only electrical; N-PCB, non-penetrative captive bolt; PCB, penetrative captive bolt. Restraining method: GP, group pen stunning; VB, V-type restraining boxes; NO, none/other.

**Table 4:** Critical evaluation of the feasibility of ABMs derived from the literature review—Physical Aspects of Feasibility. For each study, we report the study characteristics and the feasibility aspects that are directly described (e.g., the use of electroencephalogram (EEG)) or that can be derived from the information provided (e.g., seconds needed for assessing the presence of rhythmic breathing or the need to touch the animals).

ABMs	References	Time	Position	Distance	Access	Equipment	Observational Constraint
Breathing	[30]	Un	Un	Un	No	Un	Un
	[20]	Un	Un	Un	No	Yes	Un
	[31]	4–8 s ^1^	Un	Un	No	Yes	Un
	[32]	4–8 s ^1^	Un	Un	No	No	Un
	[33]	Un	Un	Un	No	Un	Un
	[34]	4–8 s ^1^	Un	Un	No	No	Un
	[9]	Un	Un	Un	No	Yes	Un
	[35]	Un	Un	Un	No	Yes	Un
Posture	[33]	Un	Un	Un	No	Un	Un
Tonic–clonic seizure	[30]	Un	Un	Un	No	Un	Un
	[31]	Un	Un	Un	No	Yes	Un
	[32]	Un	Un	Un	No	Un	Un
	[34]	Un	Un	Un	No	Un	Un
	[9]	Un	Un	Un	No	Yes	Un
	[35]	Un	Un	Un	No	Yes	Un
Corneal reflex	[30]	Un	Un	Un	Yes	No	Un
	[31]	Un	Un	Un	Yes	Yes	Un
	[32]	Un	Un	Un	Yes	No	Un
	[33]	Un	Un	Un	Yes	No	Un
	[34]	Un	Un	Un	Yes	No	Un
	[9]	Un	Un	Un	Yes	Yes	Un
	[35]	Un	Un	Un	Yes	Yes	Un
Palpebral reflex	[30]	Un	Un	Un	Yes	No	Un
	[20]	Un	Un	Un	Yes	Yes	Un
	[33]	Un	Un	Un	Yes	No	Un
Spontaneous blinking	[31]	Un	Un	Un	No	Yes	Un
Muscle tone	[32]	Un	Un	Un	No	Un	Un
	[33]	Un	Un	Un	No	Un	Un
Eye movements	[32]	Un	Un	Un	No	Un	Un
	[33]	Un	Un	Un	No	Un	Un

Physical aspects of feasibility: Time needed for the evaluation in seconds; Un, unknown. Position of the assessors to the animal; Un, unknown. Distance between animal and assessor in meters; Un, unknown. Access to the animal; Yes/No. Need for special Equipment: Yes/No; Un, unknown. Observational constraints: Yes/No; Un, unknown. ^1^ Range of time needed to observe the movements of the flanks due to the number of breaths/min in sheep (15 to 25 breaths/min).

## Data Availability

Data is contained within the article or Supplementary Material.

## Supplementary Materials

The following supporting information can be downloaded at: https://www.mdpi.com/article/10.3390/ani13081395/s1, Table S1: Prisma_checklist_S1; Table S2: Data_sheet_S2.
